# CNC Edge Finishing of Granite: Effect of Machining Conditions on Part Quality, Cutting Forces, and Particle Emissions

**DOI:** 10.3390/ma14216496

**Published:** 2021-10-29

**Authors:** Haithem Bahri, Victor Songmene, Jules Kouam, Agnes Marie Samuel, Fawzy Hosny Samuel

**Affiliations:** Department of Mechanical Engineering, École de Technologie Supérieure (ÉTS), Montreal, QC H3C 1K3, Canada; haithem.bahri.1@ens.etsmtl.ca (H.B.); jules.kouam@etsmtl.ca (J.K.); agnesmsamuel@gmail.com (A.M.S.); Fawzy-Hosny.Samuel@etsmtl.ca (F.H.S.)

**Keywords:** granite, edge finishing, part quality, particle emission, cutting forces, surface finish

## Abstract

Edge finishing is a shaping process that is extremely important in the granite and marble processing industries. It does not only shape the edge but also makes it shiny and durable. However, this process generates dust (fine and ultrafine particles) that can have a significant impact on air quality in the workshop and can put workers’ health at risk. While environmental requirements and occupational health and safety regulations are becoming increasingly stringent, at the same time, industries must continue to produce quality parts at competitive prices. The purpose of this study was to examine the surface quality, the cutting forces, and the emission of fine (FP) and ultrafine (UFP) particles during wet and dry edge finishing of granite edges as a function of the machining parameters and abrasive grit sizes. Three machining operations were investigated: roughing, semi-finishing, and finishing, using diamond abrasives (with grit sizes 45, 150, 300, 600, 1500, and 3000). The experiments were carried out on two granites, one being black and the other white. The tested spindle speeds ranged from 1500 rpm to 3500 rpm and the feed rates from 500–1500 mm/min. It was found that roughing operations produce more fine particles while finishing operations produce more ultrafine particles. These particle emissions, as well as the part quality and the cutting forces are strongly dependent on cutting speed and on the grit size of the abrasive used.

## 1. Introduction

Grinding or polishing is an operation treating the surface of a stone that gives it its final utility. At the end of this treatment, the finished surface is judged by several important characteristics, including mechanical and aesthetic aspects [1]. The grinding operation is carried out using multiple abrasive tools of different grit sizes. In the roughing phase (beginning of the grinding operation), large grit abrasives are used, while small grit abrasives are used in the finishing phase (end of the polishing operation). Xu et al. [2] defined two mechanisms of material removal in granite during the grinding process: in the roughing phase, the material removal mechanism is by brittle fracture, whereas in the finishing phase, the material shows a ductile material removal mechanism, that is, the ground surface exhibits ductile flow.

In the particular case of granite, its grinding/polishing produces crystalline silica dust, which after inhalation for long periods of time, can cause alveolar inflammation and several respiratory diseases such as silicosis and lung cancer [3]. Between 2004 and 2008, CNESST recognized 157 cases of silicosis disease [4]. The issue here is very serious because in the US, it was estimated that more than two million people are exposed to crystalline silica dust in their workplace [5]. That is why granite industries must ensure that during a five-day work week (8 h/day), workers’ exposure to quartz does not exceed a level of 0.05 mg/m^3^ (OSHA, 2016) [6].

Granite consists of quartz, feldspar, micas, amphiboles, and a mixture of additional trace minerals. These minerals and their variation in abundance and alteration give granite the numerous colors and textures observed in nature. Granite typically contains 20–60% quartz, 10–65% feldspar, and 5–15% micas (biotite or muscovite). Two types of granite, white and black, were investigated in the present study. White granite is a granite that is composed primarily of quartz (milky white) and feldspar (opaque white) minerals. Small black specks in white granite are likely small amphibole grains. The white granite used in our study consists of 41% of quartz, 33% of plagioclase, and 23% K-feldspar. The black granite is an anorthosite with coarse grains, consisting mainly of plagioclase (about 83%). It does not contain quartz. The grain size of black granite (0.2 to 17 mm) is greater than that of white granite (0.5 to 7 mm), as determined from mineralogical analysis [5]. The average density of white granite is about 2.7 g/cm^3^, while black granite has a higher average density, of about 3.1 g/cm^3^.

Studies have shown that during the grinding and polishing of granite, there are particle emissions of micrometric and nanometric sizes, but from a toxicity point of view, ultrafine particles have been shown to be more toxic than fine particles [7]. Some investigations have been carried out on the emission of fine and ultrafine particles as well as on part quality during the surface polishing of this material. It was found in the literature that, increasing the spindle speed resulted in high generation and dispersion of microparticles, with high concentrations located far from the particle generating zone [8], whereas such emissions are decreased by high feed rates [9]. Kouam et al. [10] found that during granite polishing, the emission of fine and ultrafine particles and the quality parts are highly influenced by the concentration of quartz in granite and the cutting conditions. They also showed that the use of high cutting speeds results in the generation of finer particles and a better polished surface than those obtained at low speeds.

Songmene et al. [5] studied the effect of minimum quantity lubrication (MQL) at different water flows on fine and ultrafine particle emissions during granite polishing. They found that the use of water in MQL mode was very effective in reducing the emission of fine particles (20–90%) but did not reduce ultrafine particle emissions.

Yilmaz (2016) showed that productivity and efficiency are the two major concerns in the field of stone machining [11]. In his study conducted on circular sawing of a granite workpiece, he examined the distribution and size of the chips generated as a function of productivity and efficiency parameters, such as material removal rate Q_w_. Increasing this parameter through higher depths of cut and feed rates resulted in larger chips. An increasing number of these chips in a sawing operation could be a sign of improved process efficiency.

Several studies have been conducted on the deburring and edge machining of metal parts [12,13]. Sai et al. succeeded in identifying the optimal machining parameters for the formation of the smallest burr [14]. It was found that using a 2 mm deburring tool diameter with 4000 rpm spindle speed, 100 mm/min feed rate, and 100 µm depth of cut resulted in the least burr formation during experiments. Nevertheless, particle emissions during these operations have not yet been realized, either on metal or on stone materials.

Moreover, it has been established that most of the studies on granite polishing have focused on the surface polishing method, while no research work has been reported on edge finishing. The purpose of this paper is to understand the effects of edge finishing parameters including spindle speed, feed rates, lubrication, granite type, and grain size on the part quality and emission of particles; the main objective of this study being to simultaneously ensure a good part surface finish while lowering dust particle emission.

## 2. Materials and Methods

**Figure 1:**
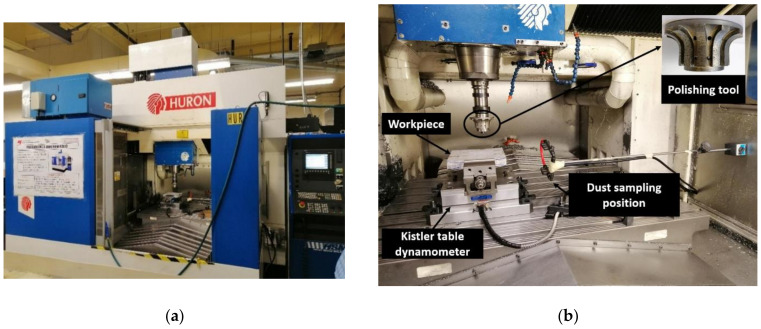
Experimental environment: (**a**) CNC grinding machine, (**b**) experimental setup.

The edge finishing of granite edges was carried out automatically on a 3-axis CNC machine (Huron K2 × 10, HURON GRAFFENSTADEN SAS, Eschau, France) as shown in Figure 1a. This machine is equipped with a dust extraction system, an oil mist extraction system, and a micro-spraying system. It has a spindle speed of up to 28,000 rpm developing a power of 40 kW, a torque of 50 N·m, and a feed rate of up to 30 m/min. Figure 1b presents the experimental setup used in greater detail.

**Figure 2:**
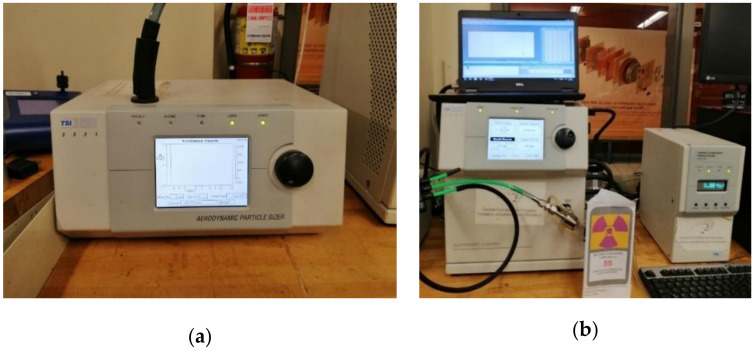
Particle acquisition systems: (**a**) APS, (**b**) SMPS.

The acquisition of micrometric particles ranging from 0.5 to 20 μm was carried out using an Aerodynamic Particle Sizer (APS) spectrometer (APS, model 3321, TSI Inc., Shoreview, MN, USA) as seen in Figure 2a, while the Scanning Mobility Particle Sizer (SMPS) (SMPS, model #3080, TSI Inc., Shoreview, MN, USA) measured the size distribution of nanometric particles as displayed in Figure 2b. This device (SMPS) can capture particles from 2.5 to 1000 nm diameter, but in this study, we limited the range between 2.5 and 333 nm. The time duration of collecting the particles was set at 50 s for both APS and SMPS equipment in all tests performed on granite pieces. At the end of the sampling time, the APS and SMPS provided data consisting of particle concentrations (numbers, mass, and surface) as a function of the particle diameters and the total mass concentration recorded.

**Figure 3:**
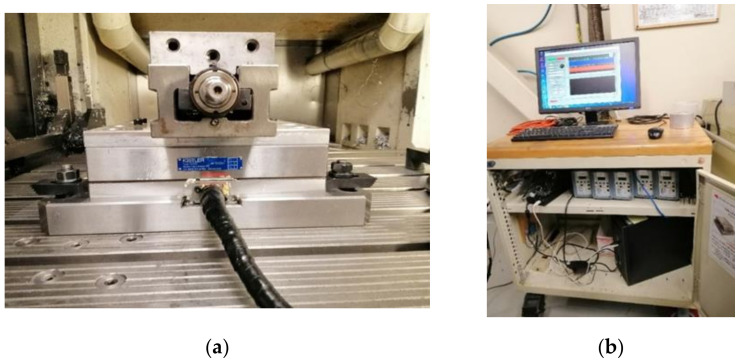
Cutting forces measurement system: (**a**) Kistler table, (**b**) Acquisition and analysis unit.

The measurement of the cutting forces was performed using a Kistler 9255B table dynamometer (Kistler Instrument Corporation, New York, NY, USA), (Figure 3), connected to a data acquisition and analysis. This device measures the cutting forces along the three axes *x*, *y*, and *z*.

The tools used were purchased from GranQuartz Canada Inc., Stanstead, QC, Canada. They consisted of six shaped tools with different grit sizes (Grits 45, 150, 300, 600, 1500, and 3000), which transformed the sharp edge of the granite sample (i.e., work part) into a rounded edge with 10 mm radius and gave a polished and shiny appearance to the part. In general, grit sizes used with respect to the three stages of a grinding/polishing operation were as follows:

Roughing: grit 45 and 150 (metal base, other alloys, large diamonds).

Semi-finishing: grit 300 and 600 (bronze alloy base, increasingly fine diamonds).

Finishing: grit 1500 and 3000 (aluminum core with rubber attached, resin layer having very fine diamond).

Table 1 gives examples of the tools used in each stage and the details.

Table 2, provided by the tool supplier (GranQuartz, Stanstead, QC, Canada), shows the maximum permitted cutting parameters (speed and feed rate) for each tool during edge finishing of granite. These limits are the maximum cutting conditions that should not be exceeded. For comparison purposes the following speeds and feed rates were used on all the tested tools:

Spindle speeds (rpm): 1000, 1500, 2000, 2500, and 3500.

Feed rates: (mm/min): 500, 1000, and 1500.

**Figure 4:**
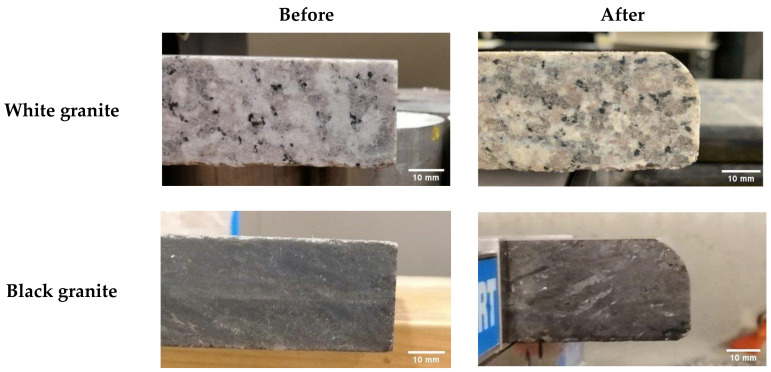
White and black granite samples before and after edge finishing.

**Figure 5:**
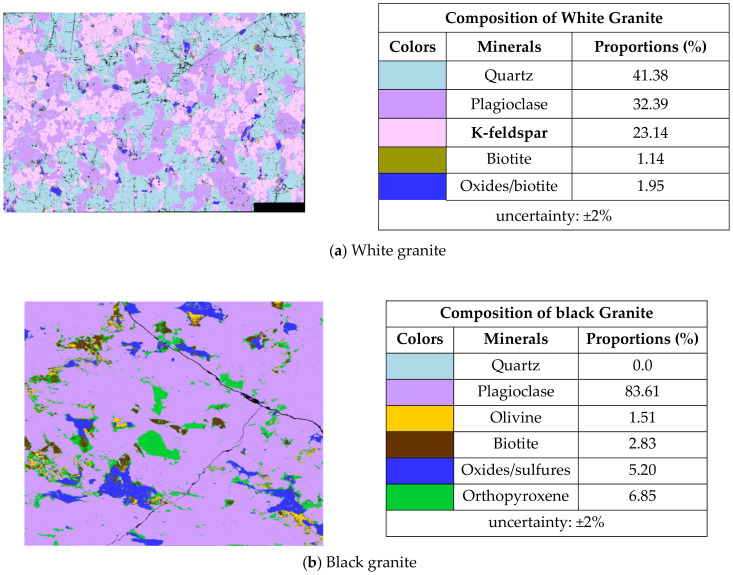
Mineral distribution of white and black granite samples obtained from SEM analysis [5].

The samples used were white and black granite squares measuring 200 × 200 × 30 mm^3^, donated by A. Lacroix Granit, Saint-Sébastien-de-Frontenac, QC, Canada. The granite workpieces were cut from these samples. Their shapes before and after the process are shown in Figure 4. It should be mentioned that the characteristics and crystallographic composition of these two materials are different. In the study of Bahloul et al. [16], scanning electron microscopic (SEM) analysis of white and black granite revealed that white granite contained 41% quartz with grain sizes ranging from 1 to 5.5 mm and the other main elements were plagioclase and K-feldspar with sizes ranging from 0.5 to 7 mm. The black granite contained no quartz but rather 83% plagioclase with grain sizes ranging from 0.2 to 17 mm. Figure 5 provides a pictorial representation of the compositions.

The total number of samples used was 11, of which 6 were white granite and 5 black granite. Lubricated grinding was done on 5 white and 5 black granite samples, while dry grinding was done on only one white sample, as the tools available did not support dry operations.

Three grinding speeds and three feed rates were studied. They were chosen based on the recommendations of the tool manufacturers. The most suitable spindle speed for all the tools is 2500 rpm which was fixed to study the effect of the feed rate while varying it from 500 to 1500 mm/min in steps of 500 mm/min (three feed rates with a center point at 1000 mm/min).

Likewise, for the grinding speed, a feed rate of 1000 mm/min was selected, being the most recommended and the spindle speed was varied from 1500 to 3500 rpm in steps of 1000 rpm, resulting in three grinding speeds with a center point at 2500 rpm.

The length of the edge to grind was 200 mm. The total grinding time for each tool varied between 8 to 24 s depending on the feed rates used. The total dust sampling time was set to 50 s. This total length was ground using 6 tools and 6 passes, one tool per pass. During each one, the particle and force sampling devices were started before the grinding and at the end of the process, a waiting time of about 1 min was observed before stopping the measuring device and then switching on the machine-tool dust suction system to flush away the dust which remained in the tool-tool.

For each sample, two opposite edges were polished. On one of the edges, the 6 tools were used over the whole length, with dust measurements made for each pass of the tool until at the end a rounded shape with a roughness of the last grit (grit 3000) was obtained; this was in addition to all the measurements of particle emission for the other grits (45, 150, 300, 600, and 1500). In the case of the roughness measurements, in order to avoid disassembling the part after each pass and wasting excessive time in setting up the machine each run, the second edge of the sample was divided into 5 equidistant slices, to get the roughness of the 5 remaining tools (45, 150, 300, 600, and 1500).

**Figure 6:**
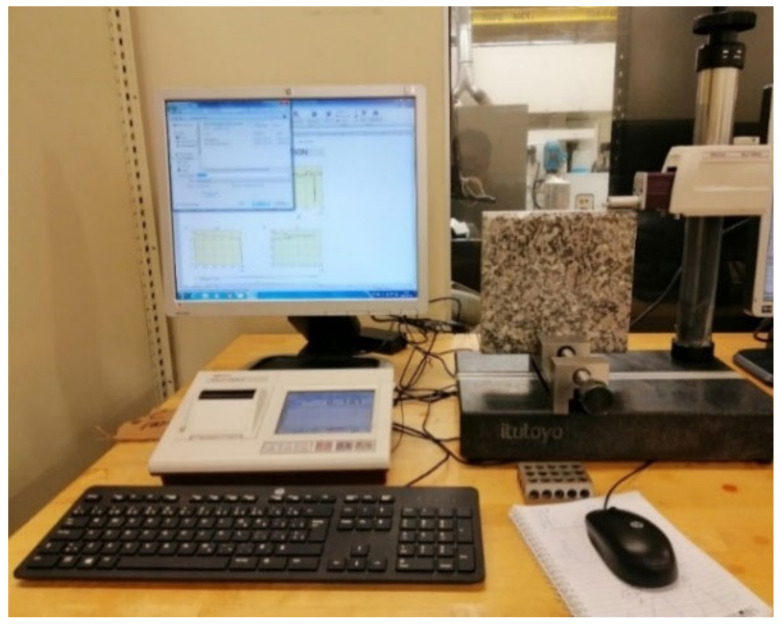
Mitutoyo Surftest SJ-410 profilometer.

Roughness parameters were measured using a Mitutoyo Surftest SJ-410 profilometer (Mitutoyo America Corporation, Aurora, IL, USA). The granite workpiece was fixed vertically on a clamp so that the probe could scan the edge surface, so that the results of the roughing parameters and the surface profile could be displayed on the computer screen (Figure 6).

## 3. Results and Discussion

It has already been shown in previous research works by the authors that the grinding of the two tested granites produces both fine particles (PM2.5) and ultrafine particles (diameters lower than 100 nm) [3,5,8,10,16]. Theses previous research works were done using rotatory grinding with exception of the work by Kouam et al. [10] which focused on ban polishing. The current research work is the first of this kind on edge finishing.

### 3.1. Fine Inhalable Particle Emission

In this section we present and discuss the result related to the emission of fine inhalable particles, sizes ranging from 0.5 microns to 10 micrometers (PM10). This range is made of inhalable particles (PM10) and fine inhalable particles, namely particulate matter of 2.5 microns in diameter and less, also known as PM2.5 or respirable particles because they penetrate the respiratory system further than larger particles [17,18].

**Figure 7:**
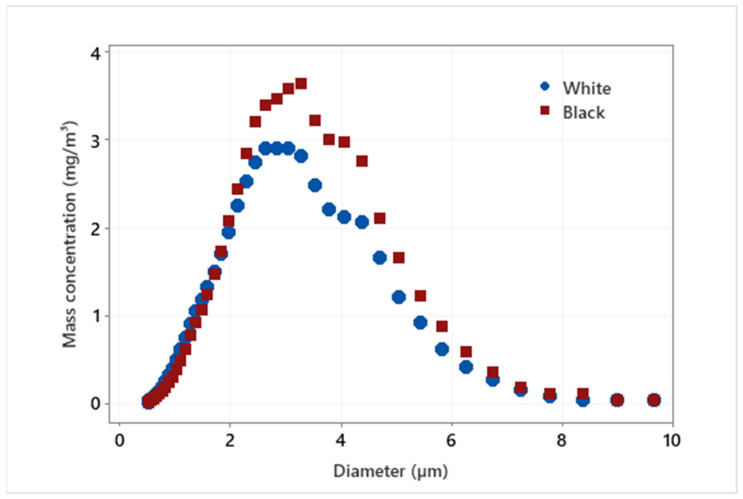
Particle size distribution of FP by mass concentration when grinding a white versus a black granite edge (Vs = 3500 rpm; f = 1000 mm/min; grit 600).

We can clearly see from Figure 7 that the largest emissions in mass concentration of fine particles of both white and black granite were with sizes between 2 and 4 µm. These particles are considered harmful as their sizes are less than 10 µm in diameter and once inhaled, they can penetrate deep into the lungs and some can even get into the bloodstream (EPA, 1987). The higher emission of FP in black granite is also notable in the figure compared to white granite.

**Figure 8:**
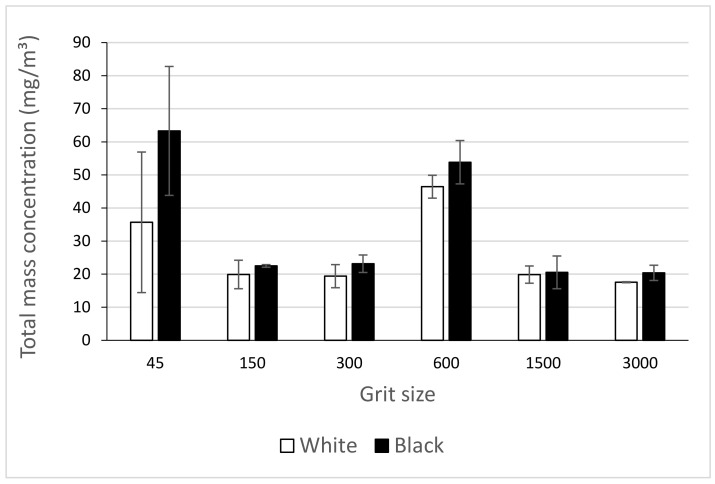
Total mass concentration of FP while grinding white and black granite for different grit sizes (Vs = 3500 rpm; f = 1000 mm/min).

The investigation of fine particle (FP) emissions during the edge finishing of black and white granite edges leads us to deduce that the generation of these particles is more significant in black granite compared to white granite (Figure 7 and Figure 8). In Figure 8, the total mass concentration of FP generated by grinding the granite edge with different grit sizes was measured by summing all the mass concentrations of each particle classified by their diameter (0.5 to 20 μm) in a table resulting from the APS data. The amount of particles emitted decreases with the decrease of abrasive grain sizes in grinding/polishing tools. This result is expected because with roughing tools there is too much material to remove so the quantity of particles is quite high, while in the finishing phase, the emission is mostly due to friction between the tool and the part, so that particles generated are smaller in size and quantity. The density and grain size of the two granite types would also affect the results to some extent.

**Figure 9:**
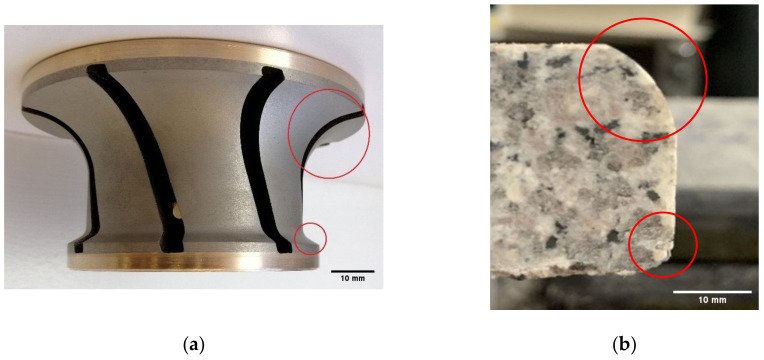
Illustration of the shapes produced with 600 grit tool: (**a**) shape on tool, (**b**) shape on granite sample.

The high emission values observed with the tool grit 600 is explained by the fact that this tool now starts to remove material at the bottom edge compared to the passage of the previous tools, which polished only the top edge with three passes of 0.1 mm depth of cut each. The tool grit 600 started the grinding of the 3 mm radius edge at the bottom, at the same time as the 15 mm radius on the top edge continued to be formed. (Figure 9).

**Figure 10:**
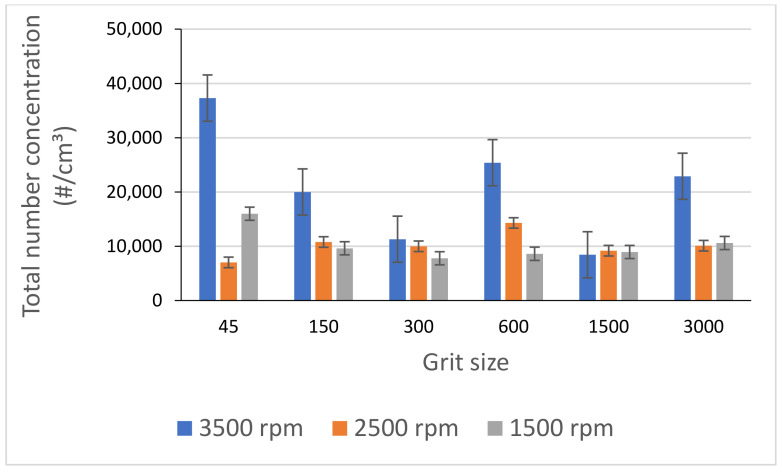
Total number concentration of FP while grinding white granite for different grit sizes (f = 1000 mm/min).

**Figure 11:**
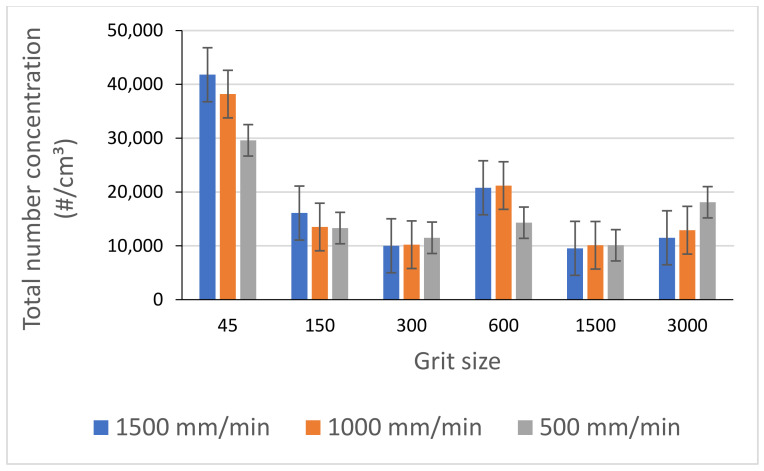
Total number concentration of FP while grinding black granite for different grit sizes (Vs = 2500 rpm).

According to the results shown in Figure 10 and Figure 11, high grinding speeds as well as feed rates are factors for large generations of fine particles. However, it cannot be deduced that the emission of these particles will decrease with the reduction of spindle speeds or feed rates. More investigations and tests are needed to interpret these behaviors.

### 3.2. Nanometric and Ultrafine Particle Emission

**Figure 12:**
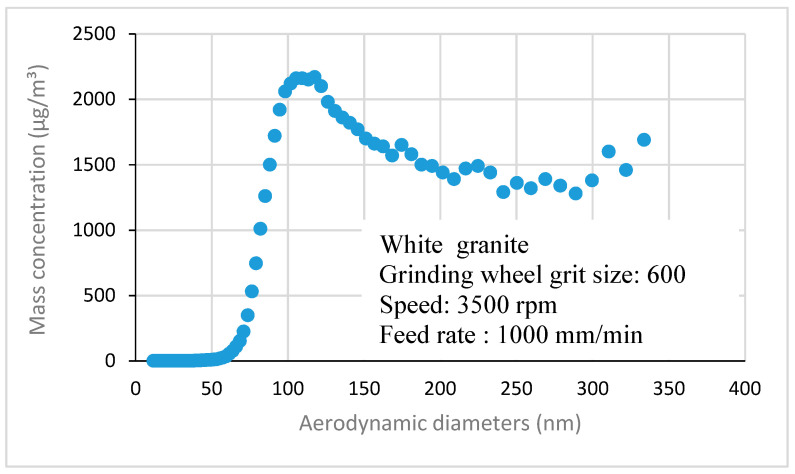
Nanometric size particle distribution obtained when grinding white granite (Vs = 3500 rpm; f = 1000 mm/min).

In this section, the results on nanometric sizes of particles emitted are presented. They consist of ultrafine particles (UFP) are particles with diameters lower than 100 nm and of particles with aerodynamic diameters between 100 and 333 nm, as shown in Figure 12. They were measured using an SMPS spectrometer equipped with a nano DMA (Differential Mobility Analyzer, TSI Inc., Shoreview, MN, USA), which enables the measurement of particles ranging from 7 to 333 nm.

**Figure 13:**
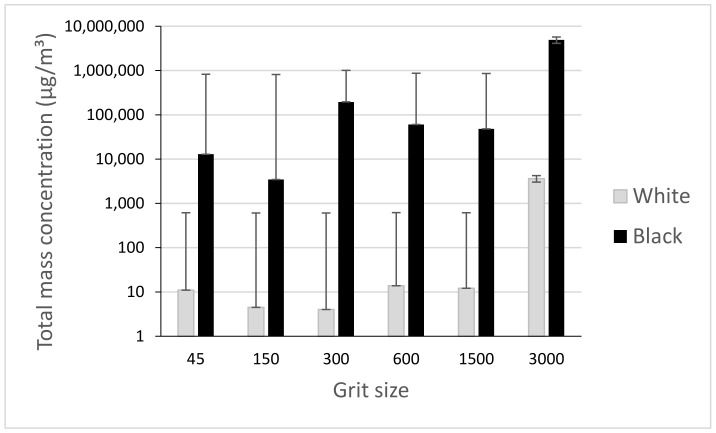
Total mass concentration of UFP while grinding white and black granite for different grit sizes (Vs = 3500 rpm; f = 1000 mm/min).

Figure 13 shows the effect of granite type on the total mass concentration of ultrafine particles (UFP). White granite, which is considered as a high-silica granite (containing ≈50% of silica), generates less UFPs than black granite, which is considered as a low-silica granite (containing ≈10% silica).

**Figure 14:**
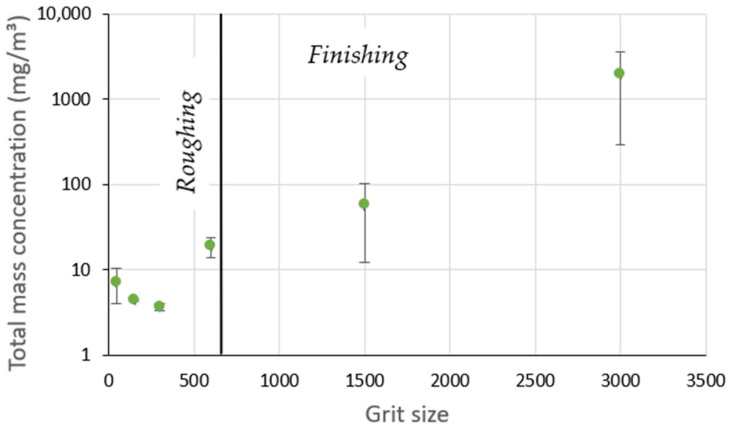
Total mass concentration of UFP while grinding white granite for different grit sizes (Vs = 3500 rpm; f = 1000 mm/min).

The effect of increasing grit sizes was studied since the use of a sequence of all the abrasive tools was mandatory in order to obtain the final roughness desired starting from a raw granite sample. As shown in Figure 14, the mass concentration of UFP goes through two phases: a roughing phase and a finishing phase. In the roughing phase, the mass concentration slightly decreases with grits up to 300. In the finishing phase with grit sizes above 300, in which there were no large amounts of material removed but only that due to friction of the surface, there was a significant increase in mass concentration of UFP.

**Figure 15:**
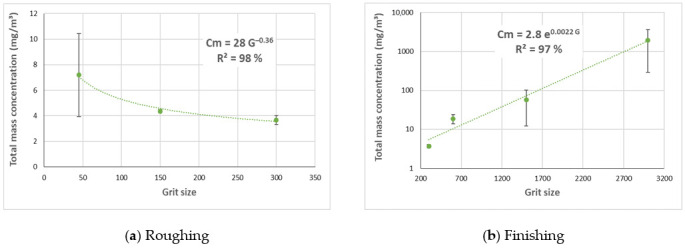
Total mass concentration of UFP while grinding white granite in two phases for different grit sizes; (**a**) roughing phase, (**b**) finishing phase (Vs = 3500 rpm; f = 1000 mm/min).

Two relationships were established as shown by Equation (1) between the mass concentration of UFP and the grit sizes depending on the grinding phase of the white granite edge (roughing or finishing) as shown in Figure 15. The correlation coefficients were high around 98% for the power relationship in the roughing phase and 96% for the exponential relationship in the finishing phase. These relationships can be written as follows:

(1) $$C_{m}=\left\{\begin{array}{ll} 28*G^{-0.36} & pour\;G\leq 300\\ 2.8*e^{0.0022*G}\;\; & pour\;G\geq 300 \end{array}\right.$$

*C_m_*: Total mass concentration of UFP [mg/m^3^]
*G*: Grit size

**Figure 16:**
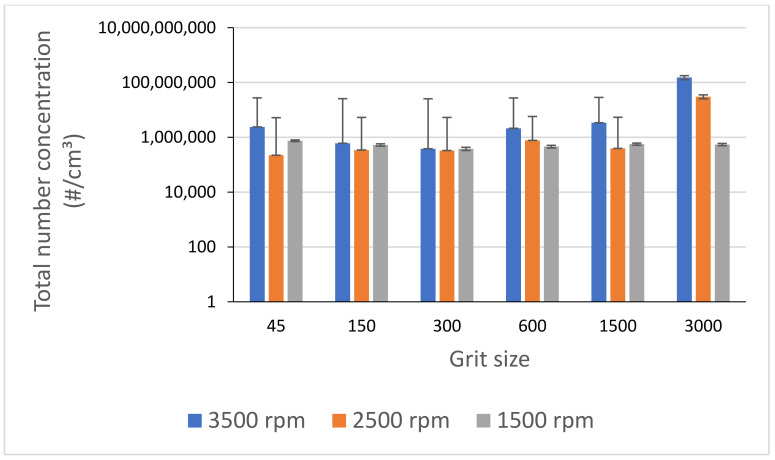
Total number concentration of UFP while grinding white granite for different spindle speeds (f = 1000 mm/min).

The use of high spindle speeds led to higher emission of ultrafine particles as shown in Figure 16, where it can be seen that in addition to the decrease in particle generation with decreasing spindle speed, the emission of particles tends to stabilize with decreasing speed.

**Figure 17:**
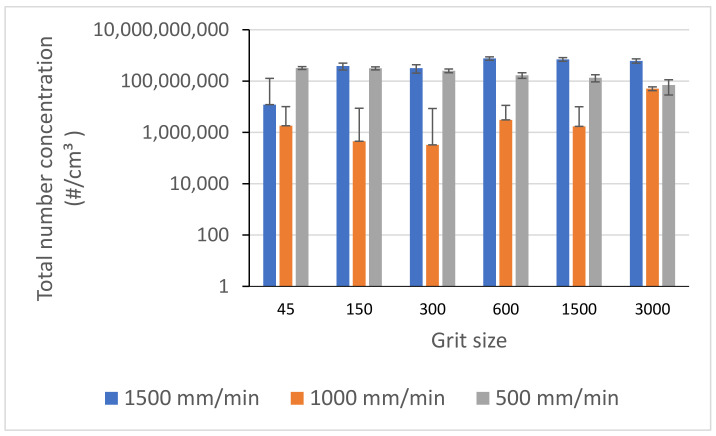
Total number concentration of UFP while grinding black granite for different feed rates (Vs = 2500 rpm).

Experiments have shown that the choice of a feed rate must be made in a judicious way, as the variation of the emission of ultrafine particles is very important from one feed rate to another. During our tests on both black and white granite, a feed rate of 1000 mm/min was identified as producing less particle emissions (FP and UFP) than the other feed rates investigated (Figure 17).

**Figure 18:**
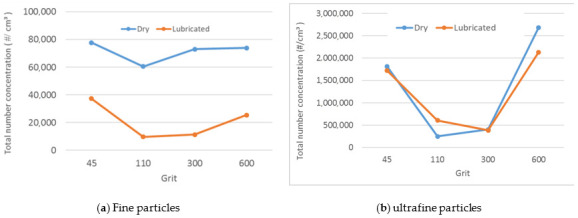
Total number concentration of particles while grinding white granite under dry and lubricated conditions: (**a**) FP, (**b**) UFP (Vs = 3500 rpm, f = 1000 mm/min, grit 600).

After finishing with the lubricated tests on the black and white granite samples, two dry tests on a single sample of white granite were made, which gave results that go well with the previous work on the effect of lubrication on the surface polishing of granite pieces [5]. It was found that the use of lubrication reduced the total number concentration of fine particles (FP) by an average of four times the concentration obtained with dry grinding but did not significantly reduce the total number concentration in the case of ultrafine particles (Figure 18).

### 3.3. Cutting Forces

Variations in cutting forces have a considerable influence on tool wear and the quality of polished parts. The evaluation of the cutting forces makes it possible to design the tools and workpiece holders, to determine the supports of the mounting in opposition to these forces, as well as to evaluate the cutting power in order to choose the right machine tool.

**Figure 19:**
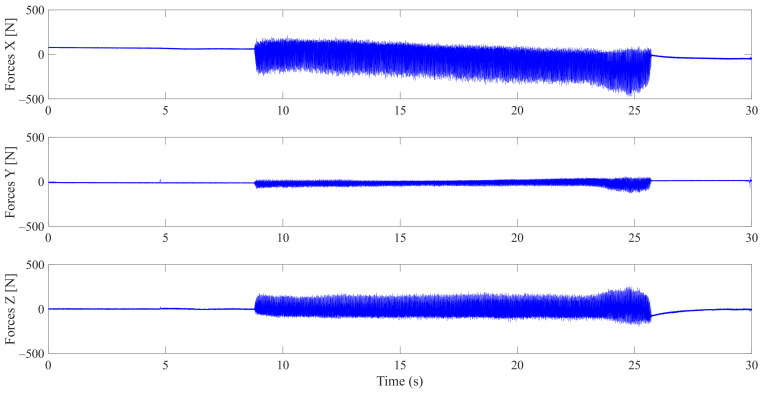
Evolution of F_x_, F_y_, and F_z_ cutting forces as a function of time during the grinding of white granite (Vs = 2500 rpm, f = 1000 mm/min, Grit 600).

The Kistler dynamometer allows the measurement of the three orthogonal components of a force. It has a high natural frequency with a very high resolution allowing the measurement of the smallest variations of large forces. Figure 19 shows the variation over time of cutting forces Fx, Fy, and Fz, for one lubricated grinding pass of a white granite edge. Fx and Fz were much larger than Fy, since the tool movement is along the *y* axis. Moreover, it can be seen that the peak forces were at the end of the grinding pass (between 23 and 28 s) where the tool heats up and friction becomes more important.

**Figure 20:**
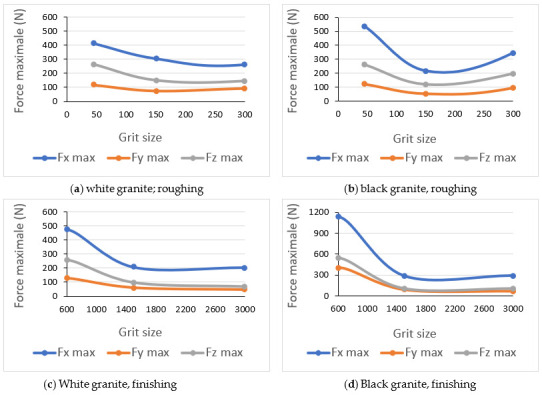
Maximum cutting forces F_x_, F_y_, and Fz as a function of abrasive grit sizes while grinding: (**a**) Roughing white granite, (**b**) Roughing black granite, (**c**) Finishing white granite and (**d**) Finishing black granite; (Vs = 2500 rpm, f = 1000 mm/min).

The maximum values of the forces along the three directions x, y, and z depending on grit sizes are represented in Figure 20a–d for both black and white granite. As we can see, the cutting forces when grinding black granite are slightly higher than those when grinding white granite. This result is in accordance with the work of Preston (1927) who demonstrated that the black granite requires more energy to polish than the white granite. This may be due to the coefficient of friction of black granite, as well as its density, which are both greater than that of white granite, but this needs to be further investigated.

The peak force observed at grit size 600 when grinding the edge of both white and black granites supports what has been previously shown in the results for particle emissions, and the fact that this tool attacks a new sharp edge at the bottom of the workpiece in addition to grinding the edge at the top.

**Figure 21:**
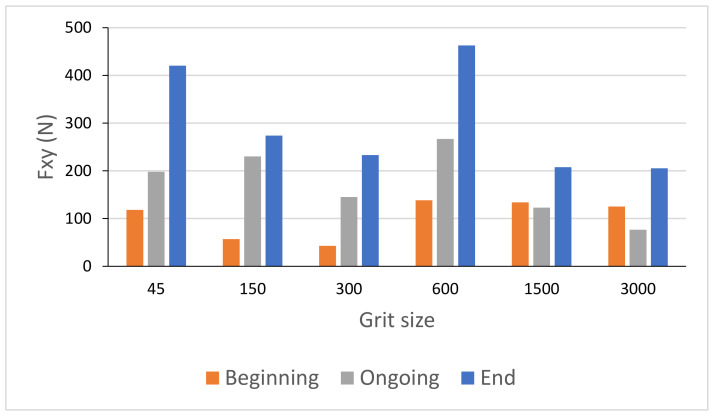
Cutting forces F_xy_ as function of grit size for white granite polished with Vs = 2500 rpm and f = 1000 mm/min.

Figure 21 shows the variation of the combined longitudinal–transversal force F_xy_ during an edge finishing operation of the granite. This force was calculated using Equation (2) where F_x_ and F_y_ were measured during three phases: in the beginning, when the tool engages the workpiece, ongoing when the tool is in the middle of the workpiece, and at the end when the tool prepares to move away from the workpiece. When using metal-based tools (grit 45 to 600), the more the tool advances in the part, the more the effort increases. This can be explained by the large amount of material removed in roughing and semi-finishing with these tools using cutting depths up to 0.1 mm, which causes tool fatigue and heating, the more the tool advances in the material. For the finishing tools (grit 1500 and 3000), high efforts were noticed when engaging and disengaging the tool from the workpiece. During this finishing phase, the cutting depth was 0.05 mm, which means that there was less material removal compared to other tools. In addition, the tool material was resistant to heat and friction but sensitive to shock, which means that in the middle of the workpiece the cutting forces were relatively lower compared to the workpiece attack and tool clearance stages.

(2) $$F_{xy}=\sqrt{F_{x}^{2}+F_{y}^{2}}$$

**Figure 22:**
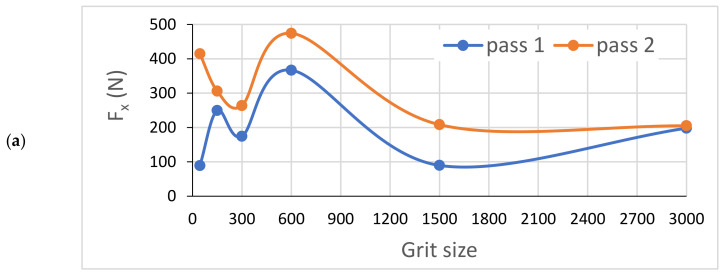

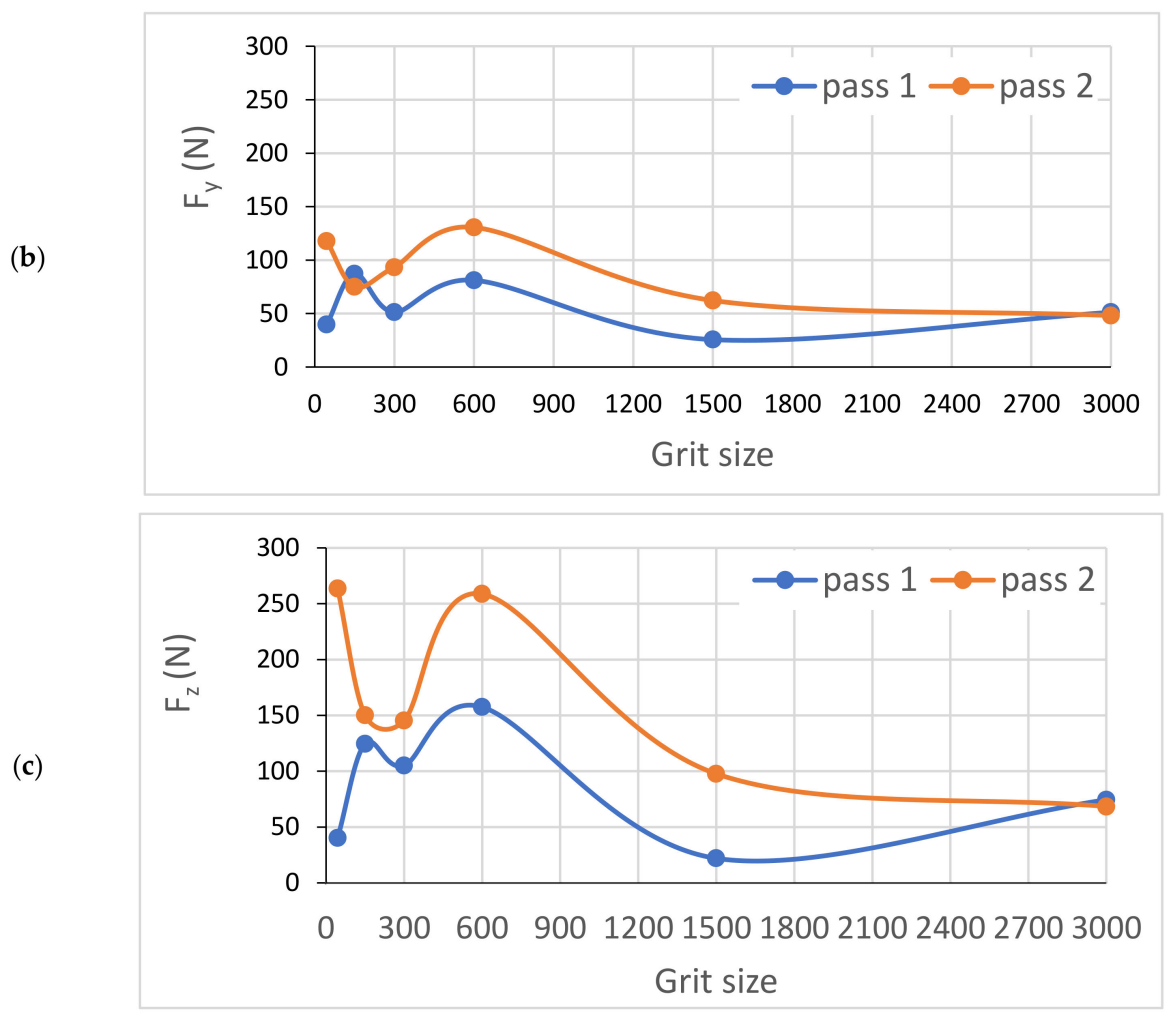
Variation of different forces while grinding white granite edge with two passes under the same conditions (Vs = 2500 rpm, f = 1000 mm/min): (**a**) Fx, (**b**) Fy, and (**c**) Fz.

In Figure 22, we can see that the cutting forces are higher when the tool passes through the workpiece the second time compared to the first one, except for the last finishing tool (Grit 3000) which had almost the same cutting forces in both passes, keeping always the same cutting depths and the same cutting conditions. Moreover, the acquisition of these cutting forces permitted the means to determine that these forces are periodic, and that their periods depend essentially on the spindle speed. This aspect could generate a more profound study on the fatigue behavior and lifecycle of tools.

### 3.4. Surface Finishing

A good surface finish of granite improves its characteristics such as brightness and abrasion resistance, which protects it from wear and chemical surface damage.

**Figure 23:**
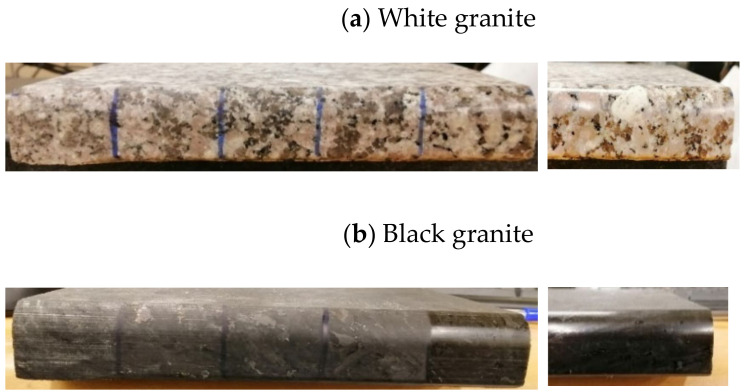

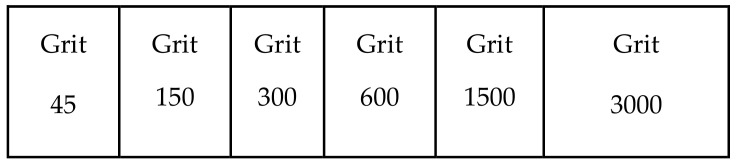
Surface finish results on white and black granite samples after wet edge finishing.

In order to see the effect of each tool with its grit size on the surface finish of the edge, Figure 23 shows the evolution of roughness in the lubricated grinding process of white and black granite edges, which clearly demonstrates the difference between the results from each tool in the two cases.

The arithmetic mean deviation of the roughness Ra was the criteria used for the comparative analysis of the surface profiles obtained after grinding.

It was seen that the increase in the tool grit size (viz., decrease in abrasive grain size) leads to a decrease in the roughness Ra, and hence therefore an improvement in the surface finish and gloss as depicted in Table 3.

**Figure 24:**
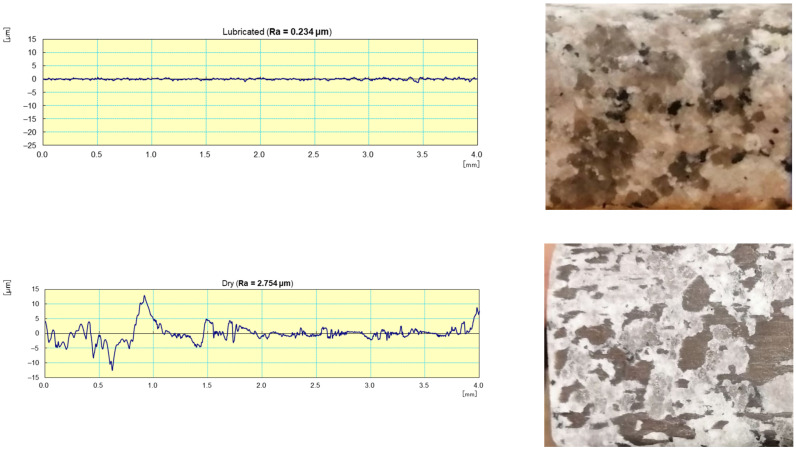
Profile surface and finish of a polished white granite edge (Vs = 3500 rpm; f = 1000 mm/min; grit 600).

With respect to lubrication, Figure 24 reveals that the use of the lubricant provided better results both in terms of roughness where the Ra for the sample subjected to dry grinding was 10 times higher than that obtained with lubrication, as well as in terms of the brightness of the edge polished (using tool grit 600).

**Figure 25:**
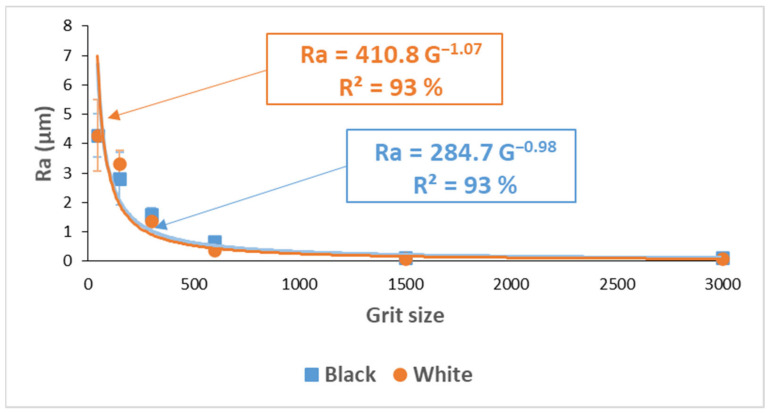
Roughness average Ra of white and black granite depending on grit size (Vs = 1500 rpm; f = 1000 mm/min).

Figure 25 displays the roughness average Ra of both types of granite at different stages of the edge finishing process with different grit sizes. This roughness parameter shows a decreasing tendency towards small values particularly with finishing tools of 1500 and 3000 grit sizes.

The relationship established between roughness and grit sizes resulting from the tendency graphs showed a correlation coefficient of 93% for both granite types. This relationship can be written as follows:

(3) $$Ra=A*G^{-1}=\frac{A}{G}$$

*Ra*: roughness average of profile measured [µm]
*A*: constant depending on the granite type. *A* = 410.8 for white granite and *A* = 284.7 for black granite.

Comparing these results with those of Saidi et al. [8] reveals that the roughness values achieved with a 1200 grit size tool in rotary polishing with vertical pressure on the workpiece, are only reached in edge finishing with a 3000 grit size tool. Moreover, the constant A in the equation for Ra was relatively different, and the correlation coefficient was lower. This may be explained by the stability and vibratory state of the part sample, which is not the same in the two cases, besides the grinding conditions implemented. In the surface polishing, the part was exposed to a vertical pressure maintained by Belleville springs and the cutting conditions were Vs = 1000 rpm and f = 500 mm/min. In the case of edge finishing, the pressure on the part was more along its horizontal axis and the cutting conditions were Vs = 1500 rpm and f = 1000 mm/min. Another point to be considered is the different grit sizes of the tools used in the two processes.

Therefore, we cannot rely on the surface finish results of the granite rotary surface polishing process to predict what will be the outcome of the edge finishing.

**Figure 26:**
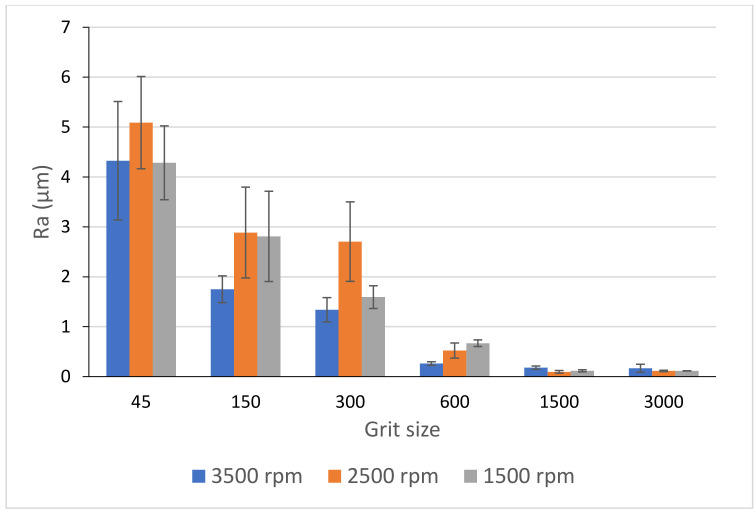
Ra-values of black granite depending on the grit size for different rotational speeds and fixed feed rate f = 1000 mm/min.

**Figure 27:**
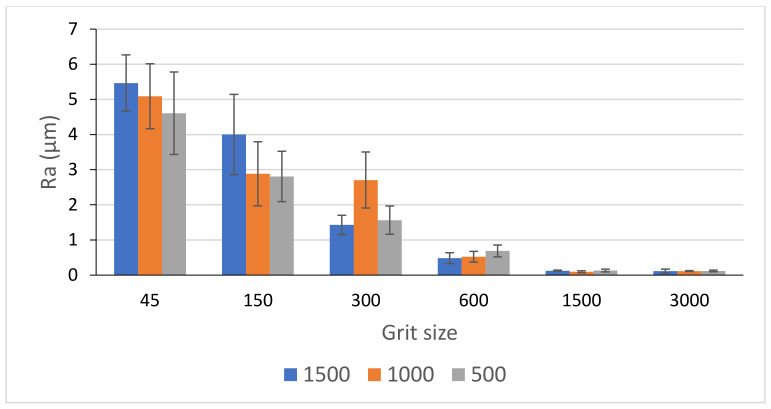
Ra-values of black granite depending on the grit size for different feed rates and fixed Vs = 2500 rpm.

Figure 26 and Figure 27 display the evolution of Ra values as a function of spindle speed and feed rate during grinding of black granite edges. A higher spindle speed leads to a better surface finish especially with roughing tools, but this does not apply to feed rate as demonstrated by Songmene et al. [19]. In fact, these two parameters did not have a significant effect on the final roughness since at the end of the process and with finishing tools, the graphs of the three spindle speeds and feed rates will converge towards very small values of Ra. Moreover, the use of all tools (roughing and finishing) in ascending order is still necessary to achieve the required surface.

## 4. Conclusions

The purpose of this research work was to study the effects of edge finishing conditions (abrasive grain size, grinding speed, feed rate, and lubrication) on part quality and dust emission (FP and UFP) in white and black granite samples. From a discussion of the results obtained, the following conclusions can be made:

1. Black granite (low silica) produces more dust (FP and UFP) than white granite (rich in silica, requiring greater energy and cutting forces in the edge finishing process.
2. Increase in tool grit size from 50 to 3000 decreases emission of fine particles but increases the generation of ultrafine particles. As the tools must be used in succession to achieve a glossy and smooth edge finishing, particle emission cannot be controlled by grit size in such cases.
3. Particle generation is affected by the grinding speed and feed rate. A combination of 1500 rpm and 1000 mm/min, while not the ideal one, is the best for reducing FP and UFP emissions.
4. In the case of roughing tools, cutting forces increase during the grinding operation, whereas with the finishing tools, large forces are only observed at the beginning and end of the operation.
5. Higher grinding speeds provide better surface finish and improve productivity. As the effect of spindle speed on roughness is significant only in roughing operations, particle emission should be focused more upon in edge finishing operations.
6. Better surface finish is obtained with the use of lubrication in the edge finishing of granite, with reduction in FP but not in UFP. Further investigation using minimum quantity lubrication (MQL) is suggested.

## Figures and Tables

**Table 1:** Details of used edge tools.

Tool Specifications	Images of Grinding Tools
Grit 45 Roughing	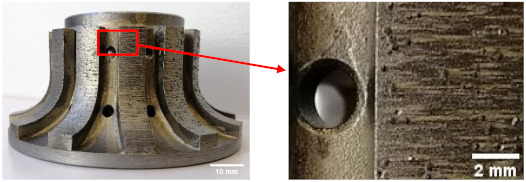
Grit 300 Semi-finishing	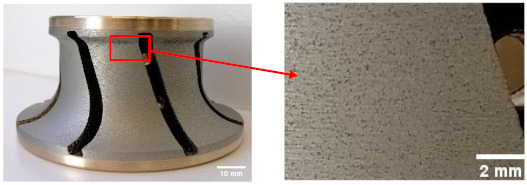
Grit 3000 Finishing	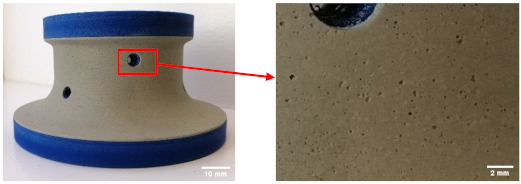

**Table 2:** Maximum cutting parameters prescribed for each edge grinding tool used (adapted from GranQuartz Canada Inc.) [15].

Tool Grit Designations	Abrasives Sizes (µm)	Speed [RPM]	Feed Rate [mm/min]
45	394	5500	1500
150	100		4000
300	49.2		3000
600	25.8	5000	1200
1500	12.6	3000	1000
3000	<8.4		

**Table 3:** Evolution of surface profiles and roughness Ra roughness of white and black granite as a function of grits (Vs = 2500 rpm; f = 1000 mm/min).

	White Granite	Black Granite
Grit 45	Ra = 6.460 µm 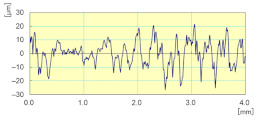	Ra = 5.089 µm 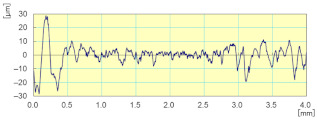
Grit 150	Ra = 3.100 µm 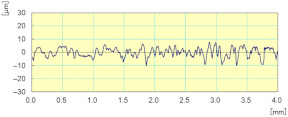	Ra = 2.885 µm 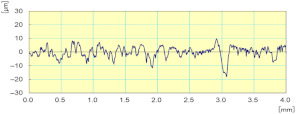
Grit 300	Ra = 1.999 µm 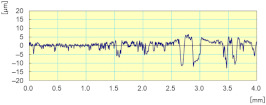	Ra = 2.705 µm 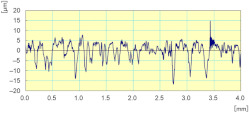
Grit 600	Ra = 0.414 µm 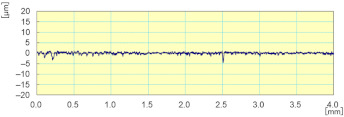	Ra = 0.522 µm 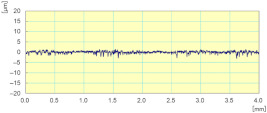
Grit 1500	Ra = 0.147 µm 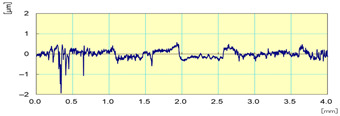	Ra = 0.114 µm 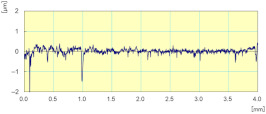
Grit 3000	Ra = 0.076 µm 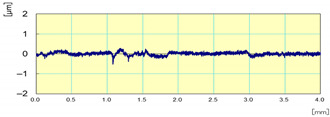	Ra = 0.096 µm 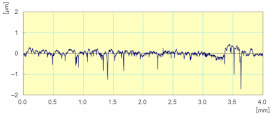

## Data Availability

Data available on request due to restrictions eg privacy or ethical.
